# Developing better strategies for school eye health screening in India

**Published:** 2017-08-07

**Authors:** Priya Adhisesha Reddy, Ken Bassett

**Affiliations:** 1 Project Manager: Aravind Eye Hospital and Postgraduate Institute of Ophthalmology, Pondicherry, India and Fulbright Scholar, Hubert H Humphrey Fellow 2016–17, Rollins School of Public Health, Emory University, Atlanta, GA, USA.; 2 Programme Director, Seva Canada and Faculty of Medicine, University of British Columbia, Vancouver, Canada.

**There is limited evidence about what is the most effective and cost-effective strategy for screening school-age children. We compare different approaches and the components that are necessary for best practice screening.**

**Figure:**
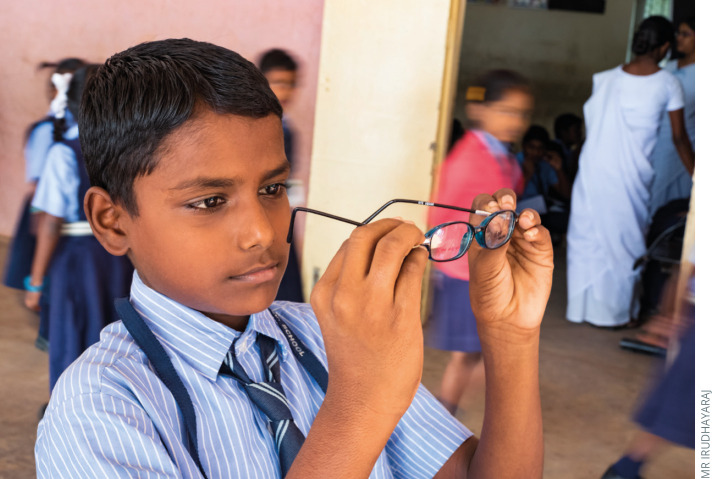
Boy choosing frame for his spectacles during school eye screening. INDIA

Good visual acuity (VA) is important for educational and behavioural development. Many countries have promoted VA screening to detect refractive errors and other ocular disorders as part of school health programmes.^1^

There is limited evidence about which strategies are cost-effective for screening visual acuity in school age children.^2^ The optimum age, number of occasions for screening, and VA screening threshold in different contexts have not been established in controlled studies.

## School-based screening

There is evidence that a school-based VA screening programme is both more effective and less costly than other primary eye care models when it comes to delivering eye care to school-going children.^3^

Due to a scarcity of ophthalmic professionals in most settings, school screening programmes have used non-eye care personnel, most commonly school teachers, who are trained to conduct the VA testing.^4^ It has been shown that teachers can be trained in VA testing with good results.^5^

## Components

Visual acuity screening includes several components:

- Examiner competence
- Awareness of students
- Examination tools
- Referral system

Each factor can influence the results of the screening test, but so far these factors have not been standardised and therefore comparison across programmes is limited. A few studies have evaluated methods within a single programme.

## Comparing selected and all-class teachers

Two studies which compared the training and use of a few selected teachers (STs) and use of ‘all class teachers’ (ACTs) or ‘more class teachers’ (MCTs) found that school screening with more teachers (i.e. fewer children screened per teacher) identified more ocular conditions requiring intervention, including refractive error. In both instances, improved accuracy was achieved without increasing the proportion of referred children.^6^,^7^

The ACT study^6^ found more true positive children with a significantly lower proportion of false positive referrals (9.7% versus 16.7%). The same study showed that the all-class teacher (ACT) model significantly increased the number of children attending follow-up within 3 months compared to the select-teacher (ST) model. This study showed that a small change in the role of teachers improved the eye care of children while reducing programme costs. Another advantage seen with ACT screening compared with STs is a shorter time between screening and follow-up examination by eye care personnel.

A major cost of school screening programmes is the time spent examining children who are referred for refraction after vision screening (the screen positives). The number of referrals is higher if vision screening is not done well (i.e., if there are many false positives) and if the screening cut-off is a relatively good level of VA, as the proportion of children in schools with this level of vision or worse will be higher.

One study examined visual acuity testing at two different VA cut-offs/thresholds.^5^ At the 6/12 cut-off (poorer VA), the number of children correctly identified as needing help (the sensitivity) was the same as at the 6/9 level (better VA). However, there were fewer false positives (i.e. unnecessary referrals), which led the authors to conclude that it is preferable to screen each eye separately at 6/12. This reduced the number of referrals by about 50% compared with a 6/9 cut-off. However, further studies are needed to determine the optimal VA cut-off in each context.

**Table 1:** Proposed school visual acuity screening preferred practices (in the Indian context)

Component	Preferred practice
Visual acuity (VA) testers	All class teachers
Training of VA testers	At school by ophthalmic professional. Give teachers a VA testing kit
Setting for screening	School: Find a quiet, private place with normal classroom lighting. Measure the distance using a 6 metre line
Process of screening	Test children one at a time; other children remain outside the room Test one eye at a time; cover the other eye with an occluder Use one row of optotypes, preferably Es, rotating it between eyes (to minimise memorisation)
Age of first examination	5 to 6 years (first year of the primary school)
Thresholds for VA testing	Cannot see 6/12 (or 6/9) Snellen line in one or both eyes
Referrals for failed VA testing	Refer children who cannot correctly identify at least 4 or 5 letters of the 6/12 (or 6/9) line with one or both eyes Refer to ophthalmic personnel, preferably seen at school within 1 month
Provision of spectacles	Provided at school within a week of refraction; usually free
Referrals to hospital	Refer all children with eye problems, regardless of their vision Refer children whose vision does not improve to normal in both eyes with refraction Give children referral cards to take to their parents. Teachers can assist with counselling/information
Compliance (spectacles + referral)	After 3 months, visit schools to find out: Do the children have their spectacles? Are they wearing them?
Frequency of VA testing	Return every 2 years if VA is normal; return every year if VA is not normal

For example, if most teaching uses blackboards, which are often of poor quality, or if classrooms are poorly lit, a better level of vision would be required for distance viewing.

## Training teachers to measure VA

In the Indian context, training teachers is typically carried out in two sessions. In the first session, ophthalmologists train teachers to recognise eye conditions in children such as squint, nystagmus, corneal opacities, ptosis, conjunctivitis and eyelid swellings. The teachers are also given posters and pictures. In the second session, optometrists instruct teachers in vision screening using eye charts. The teachers are equipped with Snellen screener charts (both number and tumbling E), 6 metre tape and data forms to record whether the child can or cannot see the optotypes with each eye. They practise on one another to standardise methods and to test reliability.

**Figure 1:**
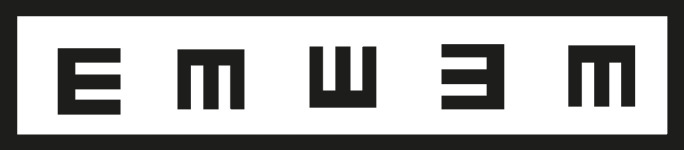
For screening, use one line of optotypes (preferably E's) at a time, with a dark surround. To download a 6/9 single-line optotype and instructions, follow the links below. http://tinyurl.com/optotype http://tinyurl.com/optotype-info

Visual acuity is measured at 6 metres using a Snellen (or E) chart, asking students to occlude one eye at a time with an occluder. To limit memorisation, and to improve reliability, a tumbling E chart can also be used. **Tip: Rotate the chart between eyes and between children.** Only one student should be examined at a time; keep others outside the examination room. During screening, students should read the cut-off/threshhold line only (6/12 or 6/9). High-contrast black on white should be used, with a dark surround, as this improves reliability when only using one row of optotypes (Figure 1).

A line is considered a pass if at least four out of five letters are read accurately. Children with spectacles should have their vision tested first without, and then with, their spectacles. An eye with visual acuity of less than 6/12 (or 6/9) is noted as ‘screening failure’ and the child is referred.^6^

In summary, the limited evidence supports school screening methods using ‘all class teachers’ and failure to see the 6/12 (or 6/9) Snellen line in one or both eyes as the referral threshold for refraction. All children suspected of having other eye problems, regardless of their vision, should also be referred for examination.
